# Oral health promotion in patients with chronic renal failure admitted in the Intensive Care Unit

**DOI:** 10.1002/ccr3.437

**Published:** 2015-11-09

**Authors:** Alexandre Franco Miranda, Erica Negrini Lia, Tatiane Maciel de Carvalho, Cinthia Gonçalves Barbosa de Castro Piau, Priscila Paganini Costa, Ana Cristina Barreto Bezerra

**Affiliations:** ^1^ Department of Preventive DentistryPost Graduation Program in Health SciencesUniversity of BrasiliaBrasiliaBrazil; ^2^ Department of Dentistry for Special PatientsCatholic University of BrasiliaBrasiliaBrazil; ^3^ Department of Preventive and Pediatric DentistryUniversity of BrasíliaBrasiliaBrazil; ^4^ University Paulista – UNIPBrasiliaBrazil; ^5^ Post Graduation in Temporomandibular DysfunctionSl Mandic CampinasSão PauloBrazil; ^6^ Department of Pediatric DentistryCatholic University of BrasiliaBrasiliaBrazil; ^7^ Department of PeriodontologyState University of LondrinaParanáBrazil

**Keywords:** Chronic renal failure, dental health service, dentistry for chronically ill patients, health care quality, intensive care units, oral hygiene, quality of life, ventilator‐associated pneumonia

## Abstract

Oral hygiene deficiency is common in patients treated in ICUs and it enables biofilm colonization by microorganisms that lead to respiratory infections. A 30‐year‐old female patient with chronic renal failure was hospitalized. Dental procedures were performed in the ICU and contributed to the patient's health after a few days.

## Introduction

Oral health promotion in intensive care units (ICUs) is considered to be a clinical practice that aims to bring health to the oral environment and quality of life to hospitalized patients. These relation with the systemic condition may be directly associated with the context of ventilator‐associated pneumonia (VAP), a disease that leads to high mortality rates in hospitals 1, 2.

This infection risk due to the fact that after three days in the hospital, especially in the ICU, there is increased bacterial colonization, particularly gram‐negative ones, associated with VAP in the oral cavity and oropharynx. Such condition reinforces the need for effective oral health practices that are able to decrease this systemic framework rate 3, 4, 5, 6.

Ventilator‐associated pneumonia (VAP) is considered to be a lower respiratory tract infection that occurs during hospitalization, since it is not present or incubated at the time the patient is admitted and affects mainly critically ill ICU‐intubated patients as well as those on mechanical ventilation 7, 8, 9.

Preventive oral health interventions conducted in ICUs aim at eliminating inflammatory, infectious, and painful symptoms that affect these critically ill individuals by removing biofilm through mechanical (lingual surface brushing and dental hygiene) and pharmacological (antimicrobial agents) actions, which may prevent the emergence of chronic systemic diseases such as pneumonia and bacterial endocarditis 2, 3, 5.

Hospitalized patients are predisposed to have poor oral hygiene because of their own systemic conditions, the medications they use, the complex medical treatments they are subjected to, their inability to perform their own oral hygiene, ICU monitoring equipment, and especially the lack of professional training and adaptation measures to oral health performing within this distinctive environment 3, 4, 8.

Studies 9, 10, 11 associate oral hygiene deficiency (biofilm accumulation and tongue coating) with VAP, since the same bacterial content found in tracheal secretions is present in the oral cavity. Such assumption emphasizes that biofilm and tongue coating may be considered to be microbial reservoirs (gram negative) associated with pneumonia.

Given these clinical conditions, the presence of tongue coating in the patient's oral cavity is understood as the most critical niche of stagnant organic matter and it leads to the formation of volatile compounds that help the occurrence of the 0.2‐mm thick coating due to the anaerobic process. This condition requires an effective oral health action in ICUs 4, 5.

Patients in intensive care units may have kidney disease and require special care. Promoting oral health – especially by eliminating inflammatory and infectious processes within the oral cavity – is essential to provide quality of life to hospitalized patients 12, 13.

The current paper aims – through a case report – to approach the oral health management and the clinical adaptation performed by a dental surgeon in a patient with chronic renal failure admitted in a hospital in Brasilia, Brazil. Project approved by the Research Ethics Committee of the Catholic University of Brasilia, Brazil, with the registration number CAAE 44578215.0.0000.0029.

## Clinical Practice – Case Report

The informed consent was signed by the family of the patient in relation to the research project and clinical case, all ethical and legal conducts were done in accordance with the Declaration of Helsinki.

**Figure 1:**
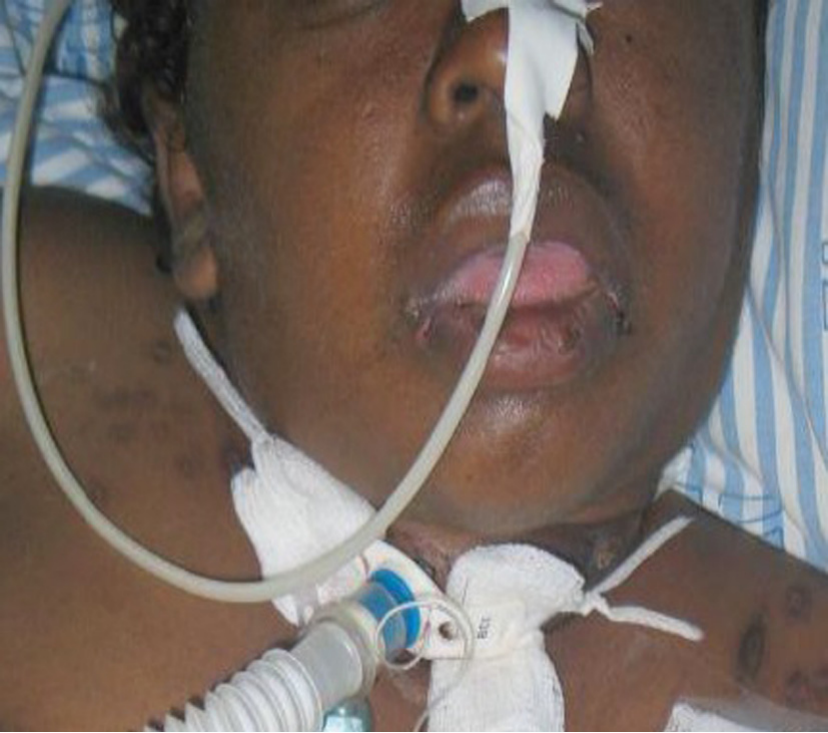
Patient female, 30 years, chronic renal failure, hospitalized for one month in the intensive care unit.

A 30‐year‐old female patient was admitted to the ICU of a hospital in Brasilia, Brazil, for life support and dialysis during 1 month. She was diagnosed with chronicle renal failure and was subjected to tracheotomy and nasogastric tube feeding. The patient was assessed by a dentist who performed oral health procedures after medical indication, since she had spontaneous bleeding in the oral cavity (Fig. 1).

After the doctor responsible for the ICU was indicated, the family signed the informed consent form in order to fulfill all the ethical and legal issues regarding the conduction of the clinical case and met all the standards in the Declaration of Helsinki.

The oral health promotion actions were planned according to the already existing routine in the ICU. These actions included assessment and planning. Clinical follow activities were adapted to the rhythm of activities performed within the ICU; however, they did not interfere in interventions from other health professionals involved with this multidisciplinary clinical context.

The patient's systemic condition was characterized as advanced stage nephropathy. She underwent hemodialysis three times a week in the ICU, and it determined that the oral health procedures were more invasive when they were performed in the different periods of the treatment the patient was subjected to.

The patient's cardiac, respiratory, and nutritional conditions were constantly monitored by machines. She was also medicated with broad‐spectrum antibiotics to reduce generalized infection.

**Figure 2:**
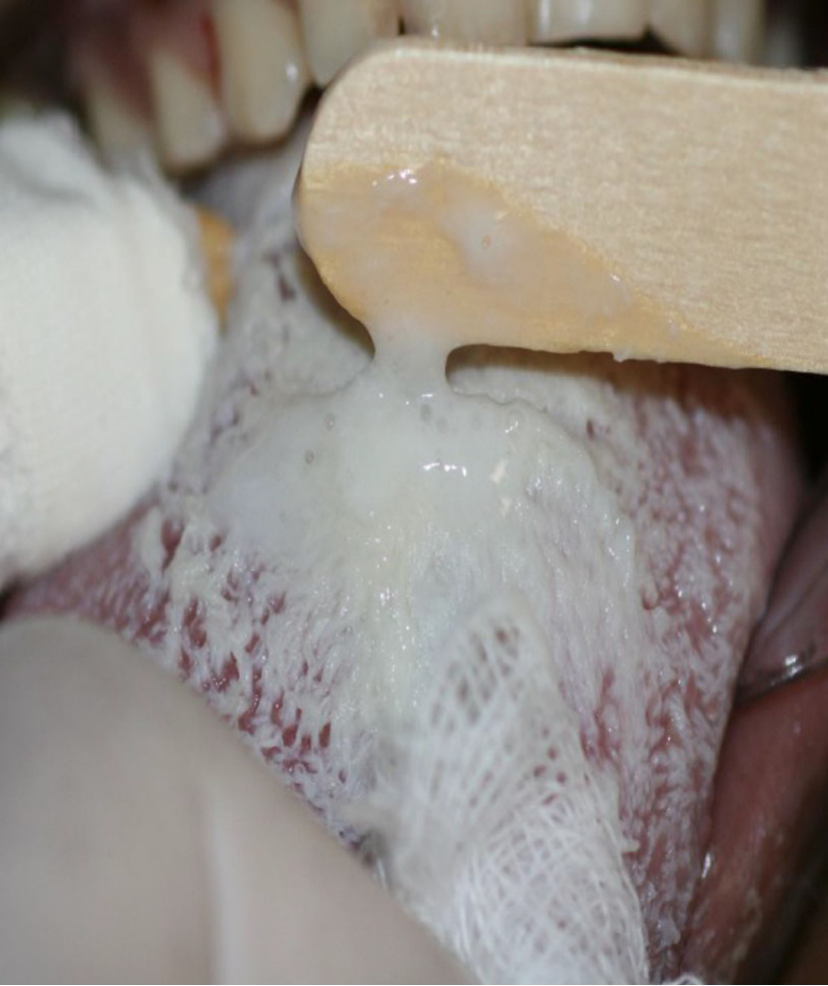
High concentration of tongue coating – oral hygiene deficiency in the intensive care unit (ICU).

At the time of oral cavity evaluation, the ICU staff members found difficulty in accessing the patient's oral cavity due to the large accumulation of tongue coating (Fig. 2), plaque and calculus – especially on the lower front teeth –, and to mouth lesions caused by dryness.

Dental care was required to improve the patient's oral health. The involvement of the physiotherapist, and/or doctor in charge, and/or nursing staff in moving the patient was requested in order to facilitate oral cavity visualization by the dental surgeon during the clinical interventions.

Every time the dental care was performed, the patient was positioned at 45°, her nasoenteral feeding was shut down to avoid heavings and vomiting, and she was under constant vacuum suction. The oral health activities were carried out using suction cannula and/or disposable surgical dental suction.

As for the handling and professional adaptation, mouth expander and mouth opener made of 12 wooden spatula, gauze and adhesive tape were used as auxiliary resources to provide a better view of the clinical performance field.

**Figure 3:**
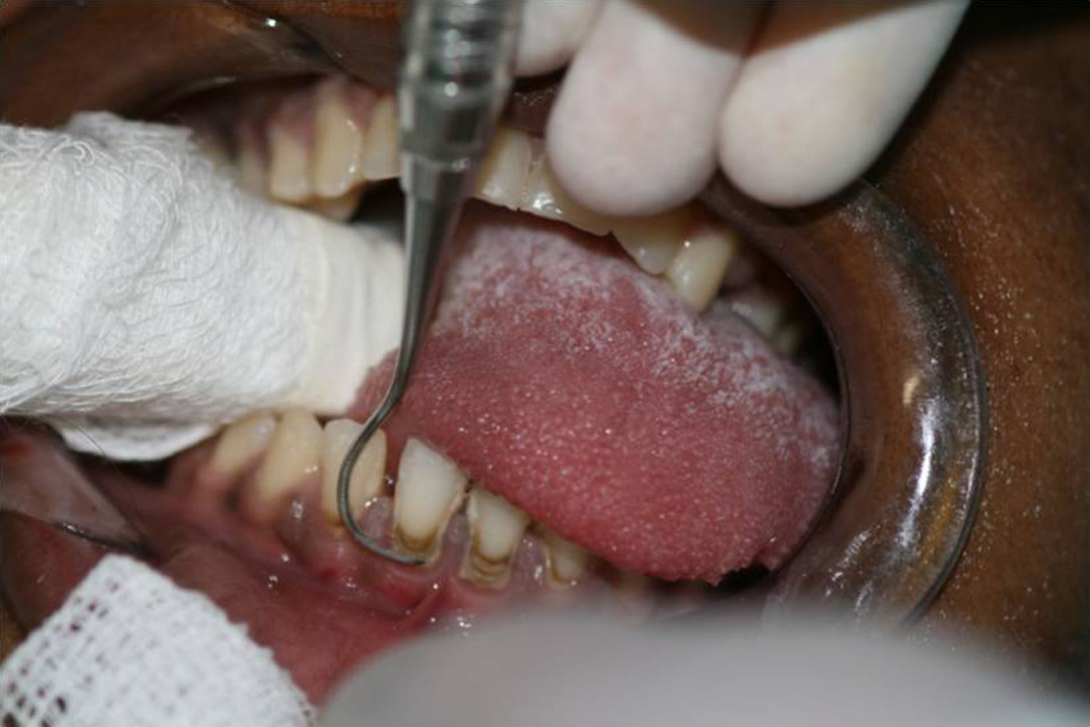
Supragingival scraping to remove calculus on anterior teeth in the ICU – Clinical conduct carried out by qualified dentist.

**Figure 4:**
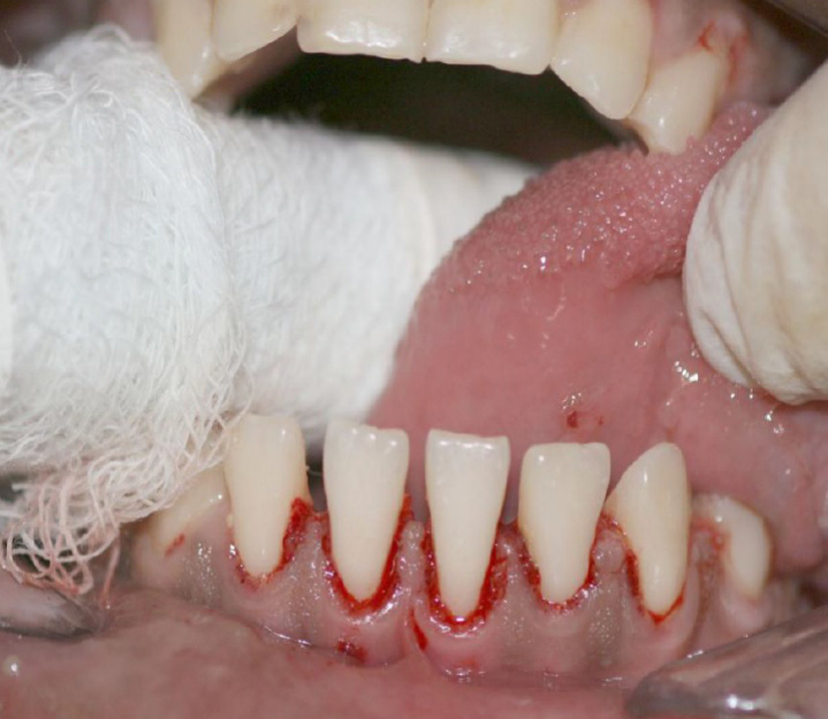
Clinical aspect after periodontal activity in ICU.

Antibiotic prophylaxis with vancomycin chloride was performed 40 min before the appointment with the dentist. It followed the correct prescription and met medical instructions. The interpapillary and gum anesthesia around the mandibular anterior teeth was performed with 2% lidocaine hydrochloride with vasoconstrictor, at 1:100 000 concentration – half of an anesthetic cartridge (1.8 mL). Periodontal supragingival scraping using McCall universal periodontal curette was applied to remove the calculus located at the vestibular (anterior) and lingual regions (posterior) of the teeth (Figs 3 and 4).

**Figure 5:**
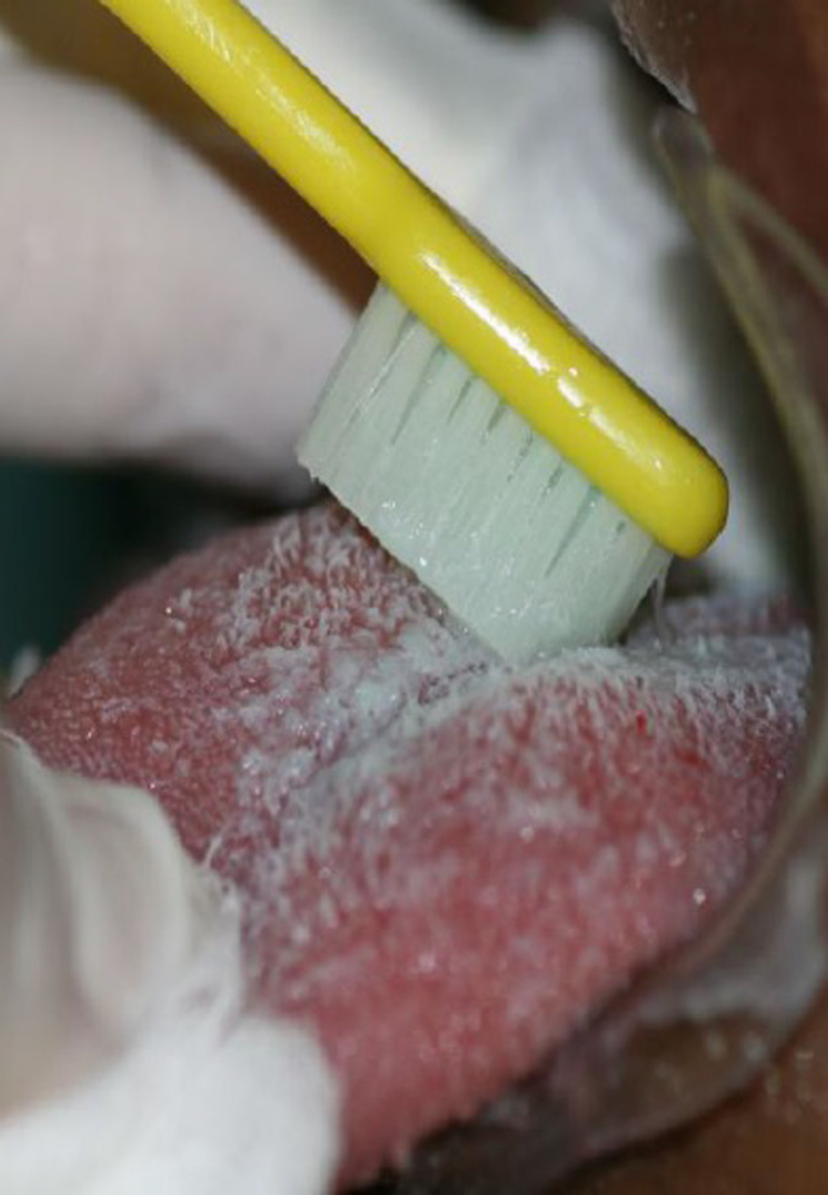
Removing tongue coating with toothbrush – posterior direction to previous.

A needle holder associated with gauze soaked in 0.12% chlorhexidine solution was used to remove the tongue coating. The hygiene was done in the forward direction. A toothbrush was also used to clean the tongue (Fig. 5).

Mechanical actions were used to remove dental plaque, and it was done under constant suction and tooth brushing. The procedure was performed using toothbrush, dentifrices, and acidulated fluoride (1.23% sodium fluoride), and it helped oral environment adequacy.

The nursing technicians and staff members were instructed about all the techniques used to remove tongue coating and dental plaque, in order to assure the patient's good clinical condition. The procedures were performed twice a day.

Guidance on the proper use of chlorhexidine 0.12% of 12–12 h, with the use of needle holder, gauze soaked in chlorhexidine, an important chemical method of reduction of biofilm, there were nurses and nursing technicians the objective of maintaining the clinical management performed, always associated with mechanical cleaning action.

Another guideline that deserves to be highlighted is the change in habits regarding endotracheal suction. It means that the suction cannula in the tracheostomy region cannot be the same that is used to aspirate organic suction in the oral and nasal cavities at the same time. The staff member was instructed to change the suction cannula in each body region to avoid cross‐contamination.

**Figure 6:**
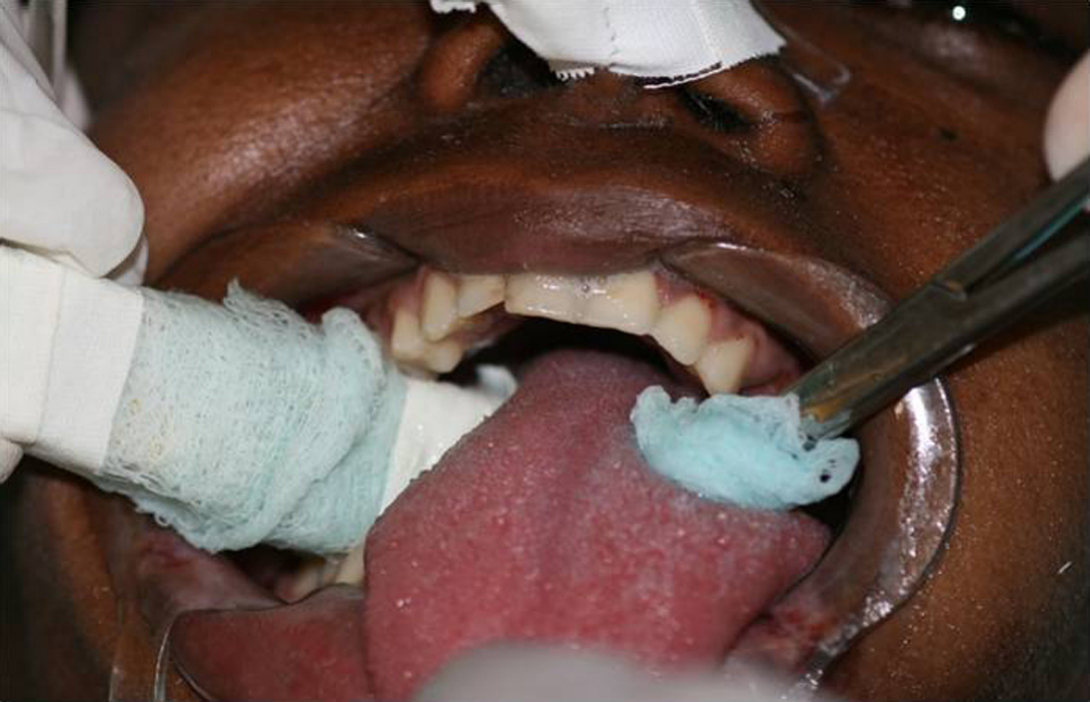
Clinical aspect after dental behaviors performed – Significant improvement of oral health of patients admitted to the ICU.

**Figure 7:**
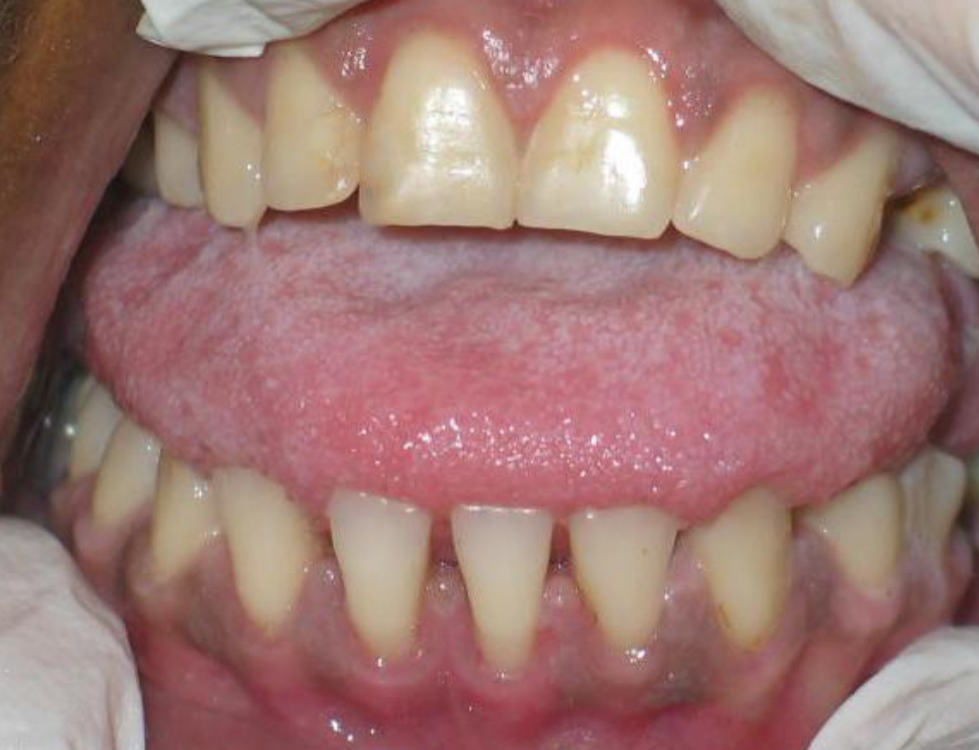
Clinical aspect after three days of activities directed to the oral health of the patient admitted to the ICU – Adequacy of the oral cavity and patient satisfaction – Elimination of inflammation, dental calculus, and tongue coating.

The patient presented satisfactory oral health condition after three days of oral health practice performed by the ICU dental surgeon and the nursing staff according to guidelines which were strictly followed by them. Thus, the microbial reservoirs that favor the emergence of ventilator‐associated pneumonia (VAP) were possibly eliminated from the patient's mouth (Figs 6 and 7).

## Discussion

Patients with chronic renal insufficiency or with progressive loss of kidney function have spontaneous bleeding in gingiva and mucosa due to blood platelet dysfunction, a fact that contributes to the poor wound healing and stronger sensitivity to future injures in the oral cavity 12, 14.

Dental care in patients with chronic renal insufficiency must be performed in the days they do not undergo dialysis, in order to avoid bleeding. Therefore, the use of heparin (anticoagulant) in these clinical activities is suggested. It is important to emphasize that, specifically in this case, the dental procedure was not performed because, according to the doctor, it was not necessary in the ICU 13, 14, 15.

In cases of invasive procedures, such as supragingival scaling, the antibiotic prophylaxis must be carried out, especially vancomycin that has less toxicity compared to other antibiotics. Vancomycin cannot be eliminated by conventional dialysis methods, is a great application of drug and convenience for patients in substitution therapy of renal function by dialysis and used in performing dental actions in critically ill patients in the ICU, according to the case report 16.

Oral health procedures, depending on the patient's clinical complexity, must be done only by dentists and health professionals who were trained to work in hospitals, mainly in intensive care units 4, 8.

The oral hygiene and the dental plaque mechanical removal are very important to prevent oral cavity diseases and systemic association. Thus, the oral hygiene involving fluoride toothpaste, tongue coating removal, and the participation of oral care professionals may contribute to significant pneumonia reduction in compromised patients in hospital (ICU) and home environments 1, 3.

The correct use of 0.12% chlorhexidine – a bactericidal and bacteriostatic agent – used daily 12 in 12 h, after oral hygiene (mechanical action) in the ICUs would prevent the formation of biofilm and tongue coating, and improve the oral hygiene conditions in bedridden and ICU patients by reducing oral colonization by gram‐negative bacteria and consequently eliminating respiratory infections. It must be always used in combined clinical conducts, in other words, prior to mechanical interventions 6, 8, 10, 11, 17.

The use of chlorhexidine 0.12% is the standard protocol of oral hygiene used in ICUs 2, 3, 4, 6, 8 to reduce the biofilm and respiratory diseases associated with mechanical ventilation. It is a microbial agent of broad‐spectrum activity against gram‐negative bacteria and has no colaterais effects for the patients 9, 10, as discussed in the report.

Dental conducts of minimal intervention, such as the proper oral hygiene using appropriate techniques for a short period of time, should be emphasized and oriented to the nursing staff who spends more time in intensive care units and is able to routinely perform these activities in ICU patients 7, 8, 9, 10.

Therefore, there is real need for the effective participation of dentists and the nursing staff in the instructions, professional qualification, and motivation of health professionals working in the ICU in order to create specific routines to promote oral health in ICU patients 7, 8, 9, 10.

The clinical procedures to promote oral health in patients with chronic renal failure have specific features and their planning should be done jointly with the entire health professional team working in the ICU. Respecting the schedules and the patient's routine in the hospital is very important 13, 15, 16.

Given this multidisciplinary health context, the dental surgeon should be a member of the team working on the promotion of health and quality of life of critically ill patients hospitalized in intensive care units, thus directly contributing to the possible recovery and/or improvement of the patients' clinical condition 4, 8, 10, 14, 17.

## Conclusions

Dental procedures, especially those requiring minimal intervention such as the correct oral hygiene protocol, must be performed at the ICUs by a trained dentist and nursing staff in order to help eliminating the potential microbial reservoirs (biofilm and tongue coating) that compromise the patients' clinical condition, as previously reported.

Trained and prepared dentists must join the multidisciplinary team working in hospitals, especially in intensive care units, in order to perform clinical training and help diagnosing hospital infections, such as nosocomial pneumonia, which is responsible for high mortality rates in ICUs.

## Conflict of Interest

None declared.
